# Two microRNAs of plasma-derived small extracellular vesicles as biomarkers for metastatic non-small cell lung cancer

**DOI:** 10.1186/s12890-023-02538-w

**Published:** 2023-07-14

**Authors:** Nan Geng, Yaopu Qi, Wenwen Qin, Si Li, Hao Jin, Yifang Jiang, Xiuhuan Wang, Shanna Wei, Ping Wang

**Affiliations:** Department of Respiratory Medicine, The Fourth Hospital of Hebei Medical University, Jiankang Road, Shijiazhuang, Hebei 050011 P.R. China

**Keywords:** Non-small cell lung cancer, Small extracellular vesicles, Gene Ontology, Kyoto Encyclopedia of genes and genomes, miRNA enrichment analysis and annotation tool, ClueGO analysis

## Abstract

**Background:**

MicroRNAs (miRNAs) of plasma-derived small extracellular vesicles (sEVs) have been proven to be associated with metastasis in several types of cancer. This study aimed to detect miRNAs of plasma-derived sEVs as potential biomarkers for metastatic non-small cell lung cancer (NSCLC).

**Methods:**

We assessed the miRNA profiles of plasma-derived sEVs from healthy individuals as the control group (CT group), NSCLC patients without distant organ metastasis as the NM-NSCLC group and patients with distant organ metastasis as the M-NSCLC group. Next-generation sequencing (NGS) was performed on samples, and differentially expressed miRNAs (DEMs) of the three groups were screened. Kyoto Encyclopedia of Genes and Genomes (KEGG) and ClueGO were used to predict potential pathways of DEMs. MiRNA enrichment analysis and annotation tool (miEAA) was used to understand changes in the tumour microenvironment in NSCLC. Quantitative reverse transcription polymerase chain reaction (qRT‒PCR) analysis was used to validate target miRNAs.

**Result:**

NGS was performed on 38 samples of miRNAs of plasma-derived sEVs, and DEMs were screened out between the above three groups. Regarding the distribution of DEMs in the NM-NSCLC and M-NSCLC groups, KEGG pathway analysis showed enrichment in focal adhesion and gap junctions and ClueGO in the Rap1 and Hippo signaling pathways; miEAA found that fibroblasts were over-represented. From our screening, miRNA-200c-3p and miRNA-4429 were found to be predictive DEMs among the CT, NM-NSCLC and M-NSCLC groups, and qRT‒PCR was applied to verify the results. Finally, it was revealed that expression levels of miR-200c-3p and miR-4429 were significantly upregulated in M-NSCLC patients.

**Conclusion:**

This study identified miRNA-200c-3p and miRNA-4429 as potential biomarkers for NSCLC metastasis.

**Supplementary Information:**

The online version contains supplementary material available at 10.1186/s12890-023-02538-w.

## Background

Lung cancer is the leading cause of cancer-related death [1]. Approximately 85% of lung cancer cases are non-small cell lung cancer (NSCLC), with histopathological types typically consisting of adenocarcinoma, squamous cell carcinoma, and large cell carcinoma [2]. In the United States, the 5-year survival rate was 24% between 2008 and 2014 for all patients with NSCLC and 5.5% for those with distant metastases [3]. Although early diagnosis and clinical treatment of lung cancer have advanced remarkably, distant metastasis is still the main cause of lung cancer-related mortality [4]. Scientists have conducted several studies on metastatic lung cancer, but the molecular mechanism underlying metastatic lung cancer has not yet been fully clarified. Therefore, it is essential to perform further research to elucidate the molecular mechanism of NSCLC and to identify new therapeutic targets for treating metastatic lung cancer.

MicroRNAs (miRNAs) are small noncoding RNAs ranging in length from 18 to 24 nucleotides. miRNAs regulate gene expression through sequence-specific binding to 3′-untranslated regions (3′-UTRs) [5], causing mRNA degradation, cleavage, and translational activation or repression [6, 7]. Increasing evidence shows that miRNAs participate in regulation of a variety of biological and pathological processes, including the occurrence and development of cancer [8]. Furthermore, multiple studies have shown that miRNAs play a role in promoting the invasion and metastasis of NSCLC [9]. However, the specific functional molecular mechanisms of miRNAs in mediating NSCLC are still elusive, and it is important to explore the potential mechanism of miRNAs.

In recent years, extracellular vesicles (EVs) have attracted much attention as important mediators of intercellular communication [10]. On the basis of their size and biological characteristics, EVs are divided into exosomes (also called small EVs), microvesicles (MVs), and apoptotic bodies [11, 12]. Messenger RNAs (mRNAs), miRNAs, proteins and lipids are complex and specific components carried by EVs [13, 14]. Delivery of these biomolecules can cause changes in gene expression in target cells. Thus, communication between cells within the tumour microenvironment (TME) by small EVs (sEVs) might play a key role in tumour occurrence, growth, metastasis, and response to therapy [15–17]. Recently, it was confirmed that miRNAs in plasma-derived sEVs are more stable and accurate than miRNAs in plasma and that they are promising biomarkers for metastasis [18].

**Fig. 1:**
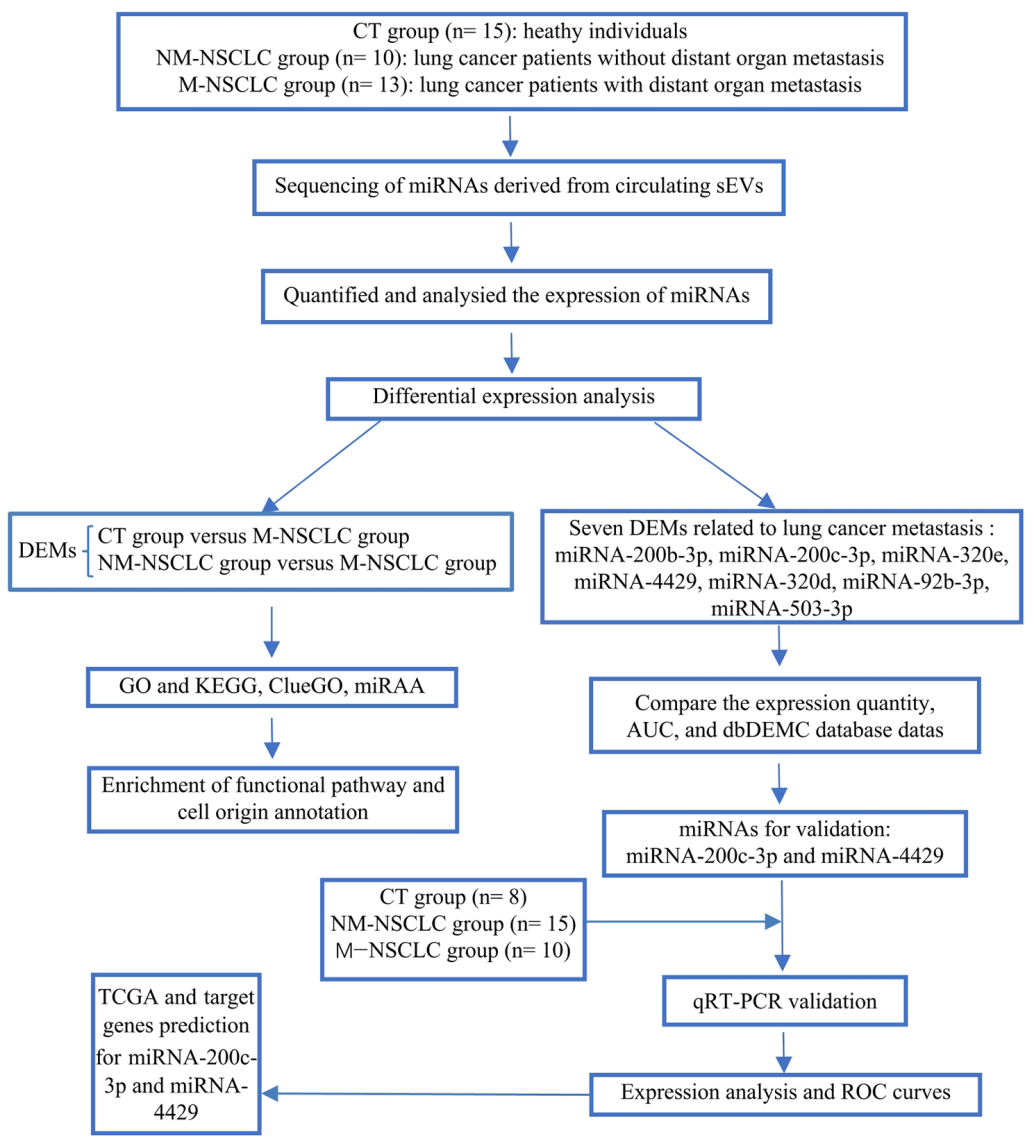
Flowchart of the study design. Abbreviations: CT, control; NM-NSCLC, nonmetastatic non-small cell lung cancer; M-NSCLC, metastatic non-small cell lung cancer; sEVs, small extracellular vesicles; miRNAs, microRNAs; DEM, differentially expressed miRNA; GO, Gene Ontology; KEGG, Kyoto Encyclopedia of Genes and Genomes; miEAA, miRNA enrichment analysis and annotation tool; AUC, area under the ROC curve; ROC, receiver operating characteristic; TCGA, The Cancer Genome Atlas; qRT‒PCR, quantitative reverse transcription polymerase chain reaction

To date, few studies have concentrated on systematic screening of plasma-derived sEV miRNAs related to metastatic NSCLC. In the present study, sEVs were isolated from plasma, miRNA sequencing (miRNA-seq) was performed, and differentially expressed miRNAs (DEMs) of healthy controls and NM-NSCLC and M-NSCLC patients were analysed. Gene Ontology (GO) and Kyoto Encyclopedia of Genes and Genomes (KEGG) pathway enrichment analyses, ClueGO enrichment analyses, and miRNA enrichment analysis and annotation tool (miRAA) were used to clarify the functions and signaling pathways of DEMs, to predict target genes and to find changes in the TME for metastatic NSCLC. These processes are all shown in Fig. 1.

Based on screening, miRNA-200c-3p and miRNA-4429 were selected as predictive DEMs and further validated in additional healthy controls and NM-NSCLC and M-NSCLC patients using quantitative reverse transcription polymerase chain reaction (qRT‒PCR). The results showed that these two miRNAs of plasma-derived sEVs can be used as biomarkers for metastatic NSCLC. Subsequently, the miRNA-seq, overall survival (OS), and clinical data for miRNA-200c-3p and miRNA-4429 in NSCLC patients were downloaded from The Cancer Genome Atlas (TCGA)-NSCLC, and a Kaplan‒Meier curve for the risk score model was used to evaluate the effect of the two miRNAs on prognosis of NSCLC. Furthermore, data for miRNA-200c-3p and miRNA-4429 were downloaded from four websites, and target genes were analysed. The intersecting genes of the four websites were selected as target genes, and the literature on the functions and pathways of target genes in NSCLC was reviewed.

In summary, this study was conducted to clarify the role of plasma-derived sEV DEMs as potential biomarkers for metastatic NSCLC and to predict potential target pathways for metastatic NSCLC.

## Methods

### Patient data and sample collection

A total of 38 participants, including 15 healthy controls, 10 patients with NM-NSCLC, and 13 patients with M-NSCLC, were enrolled in this study between August 2020 and June 2022 at the Fourth Hospital of Hebei Medical University (Shijiazhuang, China). MiRNA-seq was performed for all participants. Additionally, 8 healthy controls, 15 patients with NM-NSCLC and 10 patients with M-NSCLC were enrolled for qRT‒PCR validation. Inclusion criteria for the NSCLC patients were as follows: (1) initially diagnosed with NSCLC pathologically; (2) no history of therapy; and (3) agreed to participate in peripheral blood collection. Patients with a history of other malignant diseases were excluded. Staging of NSCLC was performed according to the National Comprehensive Cancer Network (NCCN) guidelines (version 1.2020) [19]. The healthy controls were the health examiner of the same period. Inclusion criteria for the healthy controls were as follows: (1) no history of disease and (2) availability of peripheral blood samples. All patients provided written informed consent to collect plasma samples and use their pathological data in this study. All experiments were performed in accordance with relevant guidelines and regulations. Blood samples were collected in 4 mL vacutainer tubes with EDTA anticoagulant and stored at 4 °C for no more than 2 h. The samples were centrifuged at 4 °C and 1500 × g for 10 min and then at 4 °C and 3000 × g for 15 min, and the extracted plasma was stored at − 80 °C before use.

### Isolation of plasma sEVs

In this study, size exclusion chromatography (SEC) was used to isolate sEVs from plasma [15]. According to the manufacturer’s instructions, 1 mL of 0.8 μm-filtered plasma was diluted 1.5-fold with phosphate-buffered saline (PBS) and purified with an Exosupur chromatographic column (Echo Biotech Co., Ltd., Beijing, China). Then, the samples were eluted with 0.01 M PBS, 2 mL of eluent fractions were collected, and the fractions were concentrated to 200 µL using an Amicon Ultra-15 centrifugal filter (Merck, Berlin, Germany).

### Nanoparticle tracking analysis (NTA)

The ZetaView PMX 110 kit (Particle Metrix, Meerbusch, Germany) equipped with a 405 nm laser was used to examine the vesicle suspension at concentrations between 1 × 10^7^/mL and 1 × 10^9^/mL. Then, the quantity and size of isolated particles were determined. NTA software (ZetaView 8.02.28) was used to analyse particle motion and capture a 60 s video at a frame rate of 30 frames/s.

### Transmission electron microscopy (TEM)

The sEV-enriched supernatant (10 µL) was placed on a copper mesh and incubated for 10 min at room temperature. After washing with sterile distilled water, the sEVs were treated for 1 min with a uranyl acetate solution and dried for 2 min under an incandescent lamp. Then, using transmission electron microscope (H-7650; Hitachi, Tokyo, Japan) to observed and photographed the sEVs.

### Western blot analysis

The sEV-enriched supernatant was denatured in 5× sodium dodecyl sulfonate (SDS) buffer and used for Western blotting (10% SDS-polyacrylamide gel electrophoresis; 10–30 µg protein/well). Antibodies against the following were used: CD63 (sc-5275; Santa Cruz Biotechnology, Dallas, TX, USA), HSP70 (ab181606; Abcam, England), TSG101 (ab125011; Abcam, England), and calnexin (10427-2; Proteintech, Rosemont, IL, USA). The wet rotation method was used, and the membrane was completely immersed in 3% bovine serum albumin (BSA)-TBST and gently shaken at room temperature for 30 min. The primary antibody was diluted with 3% bovine serum albumin (BSA)- Tris-buffered saline Tween (TBST), incubated at room temperature for 10 min, and placed at 4 °C overnight. On the next day, the membrane was incubated at room temperature for 30 min, followed by washing with TBST 5 times for 3 min each time, followed by incubation with the secondary antibody. Electrogenerated chemiluminescence reagents were added to the membranes, and signals were detected by an automatic chemiluminescence imaging system (Tanon 4600; Tanon Co., Ltd., Shanghai, China).

### RNA isolation and qRT‒PCR

According to the manufacturer’s protocol, total RNA was extracted from sEVs using the miRNeasy Plasma Advanced kit (catalogue number 217,204; Qiagen, Hilden, Germany). Then, using the PrimeScript™ RT reagent kit (RR037A; Takara, Shiga, Japan) to reverse transcribe total RNA to synthesize cDNA. qRT‒PCR was used to measure target gene expression, and 2 µL of cDNA was used for each PCR. In addition, cel-39 was used as an outer reference, and gene expression levels were calculated using the 2 ^−ΔΔCT^ method. The sequences of the primers and probes are shown in Table S1.

### Library preparation and sequencing

Using a total of 10 ng RNA of each sample as the input material to prepare the small RNA library. According to the manufacturer’s instructions, used the QIAseq miRNA library kit (Qiagen) to generate the sequencing library, and added the index code to the attribute sequence of each sample. Library quality was evaluated by Agilent 2100 Biological Analyzer (Agilent, Technologies, Inc., Santa Clara, CA, USA). According to the manufacturer’s instructions, used TruSeq PE Cluster Kitv3 cBot HS suite (Illumina Inc., San Diego, CA, USA) to cluster index-coding samples on the acBot Cluster Generation system. The library preparations were sequenced using the Illumina HiSeq platform (Illumina Inc.), and terminal reads were generated.

### Quantification and differential expression analysis of miRNAs

Clean reads were filtered for small nuclear RNA (snRNA), ribosomal RNA (rRNA), transfer RNA (tRNA), small nucleolar RNA (snoRNA), and other ncRNA. Each miRNA was readed from the mapping results and was calculated the transcripts per kilobase million (TPM). Differentially expressed miRNA was considered as log2|fold change| > 0.58 and *P* ≤ 0.05 based on Edge R software analysis, and the “pheatmap” R package was used to perform hierarchical clustering.

### GO and KEGG pathway enrichment analyses

miRanda and RNAhybrid software were used to predict target genes. The screening criteria for the miRanda database were set to energy ≤ -20 and score ≥ 150, and those for the RNAhybrid database were set to energy ≤ -25 and P < 0.05. GO enrichment and KEGG pathway analyses were carried out to analyse the key functions and pathways of target genes [20–22]. GO terms and KEGG pathways were determined by Fisher’s exact test and the χ^2^ test, and the threshold of significance was set as P < 0.05.

### ClueGO enrichment analysis

ClueGO was applied to analyse different pathways for different groups and present data related to the localization of hub genes. The results were automatically mapped on the network in different visual styles [23]. With Benjamini‒Hochberg correction, the statistical significance for functional enrichment analysis was set as P < 0.045, and a kappa score of 0.5 was considered in the bilateral hypergeometric test. The analysis was conducted using the DEMs of NM-NSCLC vs. M-NSCLC.

### miRNA enrichment analysis and annotation tool analysis

The miRNA enrichment analysis and annotation tool (miEAA) was used for analysis of human precursor and mature miRNAs [24]. The tool is freely accessible at https://www.ccb.uni-saarland.de/mieaa2. Either miRNA or precursors from miRBase (version 2.0) were input for miEAA. The overrepresentation analysis (ORA) method was employed, the DEMs of the NM-NSCLC group versus the M-NSCLC group were imported. The categories included cell-type specific (Atlas), immune cells, and expression profiles of genes within different tissues (Tissue Atlas). The significance threshold was set to P < 0.1, and the minimum hits per subcategory was equal to 1.

### Validation of the prognostic risk score

MiRNA expression profiles and clinical data were downloaded from The Cancer Genome Atlas (TCGA)-NSCLC (http://tcga-data.nci.nih.gov/tcga/findArchives.htm). Patients were divided into two groups using the median expression level of miRNA-200c-3p and miRNA-4429 as the cut-off value: a high-risk group with a higher expression level of miRNA and a low-risk group with a lower expression level of miRNA. The predictive values of the risk score were assessed by survival curves, sorting scatter diagrams, and distribution heat maps.

### Predicting target genes of the two miRNAs

Data for miRNA-200c-3p and miRNA-4429 data were downloaded from microRNA TargetScan (https://www.targetscan.org/vert_72/), miRDB (http://mirdb.org/), microRNA Data Integration Portal (mirDIP, http://ophid.utoronto.ca/mirDIP/), and microRNA‒target interactions database (miRTarBase, https://mirtarbase.cuhk.edu.cn/). We restricted our search by considering the microRNA TargetScan prediction as the cumulative weighted context + + score < -0.3 points and miRDB of the selected target score > 75 points; the mirDIP of the score class was very high, and miRTarBase showed no score. The target genes for miRNA-200c-3p and miRNA-4429 were considered the intersecting genes in the four websites.

### Statistical and bioinformatics analyses

Logistic regression analysis was used to assess the prognostic risk of candidate miRNAs. The area under the curve (AUC) was calculated from the receiver operating characteristic (ROC) curve to evaluate performance. R 3.5.1 (www.R-project. org) and GraphPad Prism (GraphPad Software Inc., San Diego, CA, USA) software were used for statistical analysis. Application of single factor analysis of variance (ANOVA) for comparison between groups, and P < 0.05 was considered with statistically significant.

## Results

### Clinical characteristics of the control, NM-NSCLC, and M-NSCLC groups

In this study, 15 healthy controls were enrolled and assigned to the control (CT) group, 10 patients without distant organ metastasis were assigned to the NM-NSCLC group, and 13 patients with distant organ metastasis were assigned to the M-NSCLC group. Patients in the NM-NSCLC group were at I-III stages, and those in the M-NSCLC group were at the IV stage. The metastatic sites of M-NSCLC were the brain, bone, liver and adrenal gland. The participants’ demographic and clinical data, such as age, sex, smoking history, tumour stage, pathological type, and metastatic site, are summarized in Table 1 and Table S2. There were no significant differences in age, sex, or smoking history among the three groups.

**Table 1:** Clinical characterization of participants in the CT, NM- NSCLC and M- NSCLC groups

Characteristics	Screening				Verification			
	CT group (n = 15)	NM-NSCLC group (n = 10)	M-NSCLC group (n = 13)	p	CT group (n = 8)	NM-NSCLC group (n = 15)	M-NSCLC group (n = 10)	p
Sex				P = 0.931				P = 0.672
Male	7	5	7		5	12	8	
Female	8	5	6		3	3	2	
Age	63.1 ± 6.5	62.6 ± 11.9	61.2 ± 7.2	P = 0.842	56.8 ± 8.5	64.9 ± 6.0	56.4 ± 15.2	P = 0.054
Smoking history				P = 1.000				P = 0.532
Yes	5	3	4		2	6	5	
No	10	7	9		6	9	5	
Stage								
I-III	-	10	-		-	15	-	
IV	-	-	13		-	-	10	
Pathological type				P = 0.281				P = 0.087
Adenocarcinoma	-	7	12		-	3	6	
Squamous cell carcinoma	-	3	1		-	12	4	
Metastatic site								
Brain	-	-	6		-	-	5	
Liver	-	-	0		-	-	2	
Bone	-	-	4		-	-	2	
pleura	-	-	2		-	-	0	
adrenal gland	-	-	0		-	-	1	

### Characterization of plasma-derived sEV-enriched fractions by specific screening

**Fig. 2:**
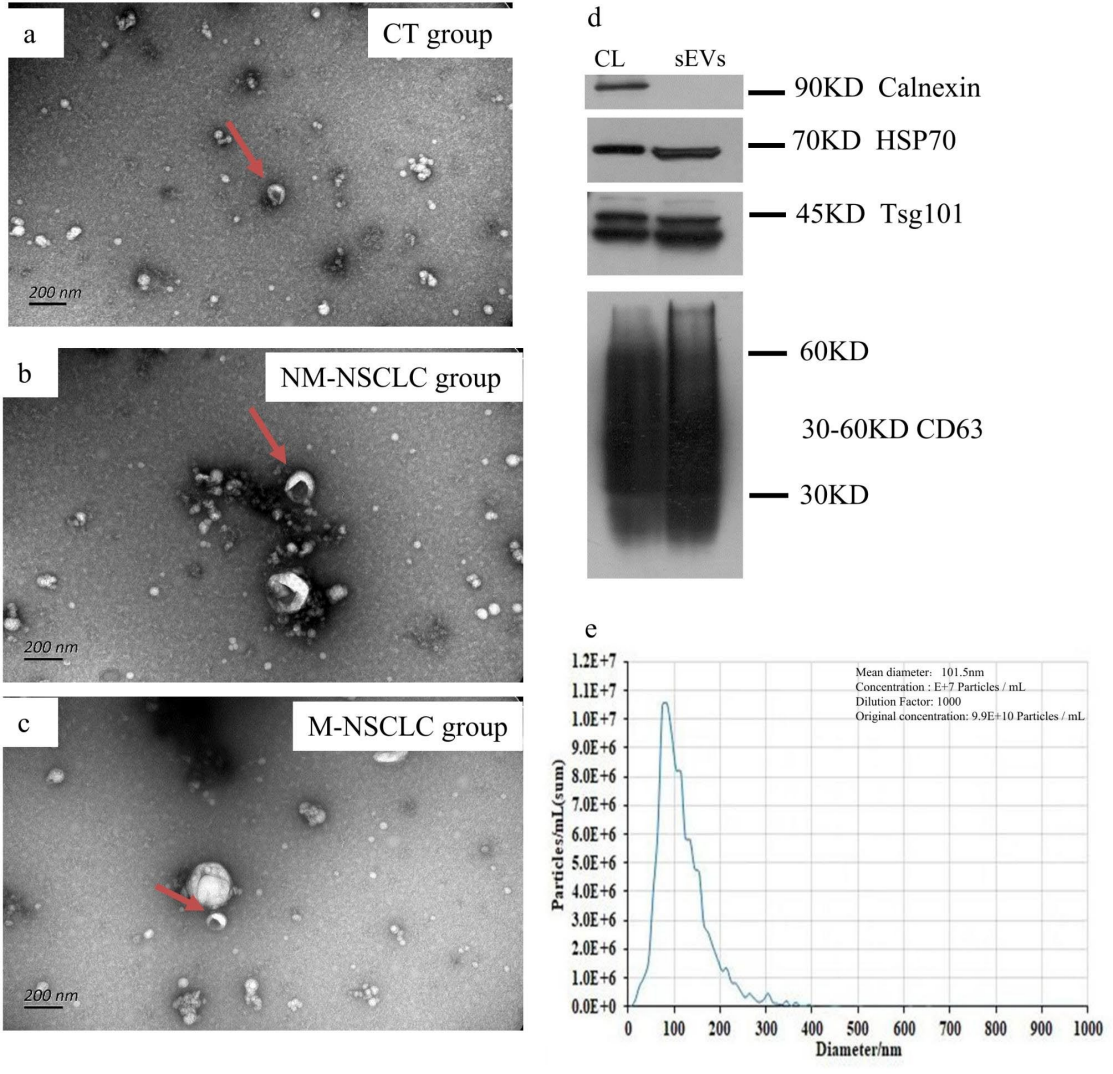
sEV-enriched fractions isolated from participants’ plasma. **a-c**. TEM images showing that sEVs are oval or bowl-shaped capsules without CT in the NM-NSCLC and M-NSCLC groups. **d**. The sEV markers Tsg101, CD63, and HSP70 were all detected in the sEV-enriched fractions isolated from the plasma; calnexin, as a negative marker of sEVs, was absent in our isolated sEV-enriched fraction samples. Abbreviation: CL, cell lysate. **e**. The NTA image shows a sample for the mean diameter and concentration of sEVs.

In this study, sEVs from plasma samples of the CT, NM-NSCLC and M-NSCLC groups were isolated using SEC and characterized by TEM, NTA, and Western blotting. TEM images showed that the sEVs of the different groups were small cup-shaped membrane vesicles (Fig. 2a-c). The isolated sEVs were highly enriched in HSP70, CD63 and TSG101 as sEV-specific markers; however, as a negative sEV marker, calnexin was not detected (Fig. 2d). NTA was applied to detect size and concentration, which showed a sample for the mean diameter and concentration of sEVs, as shown in Fig. 2e.

### Comparison of miRNA profiling and bioinformatic analysis of sEV-derived miRNAs among the CT, NM-NSCLC, and M-NSCLC groups

**Fig. 3:**
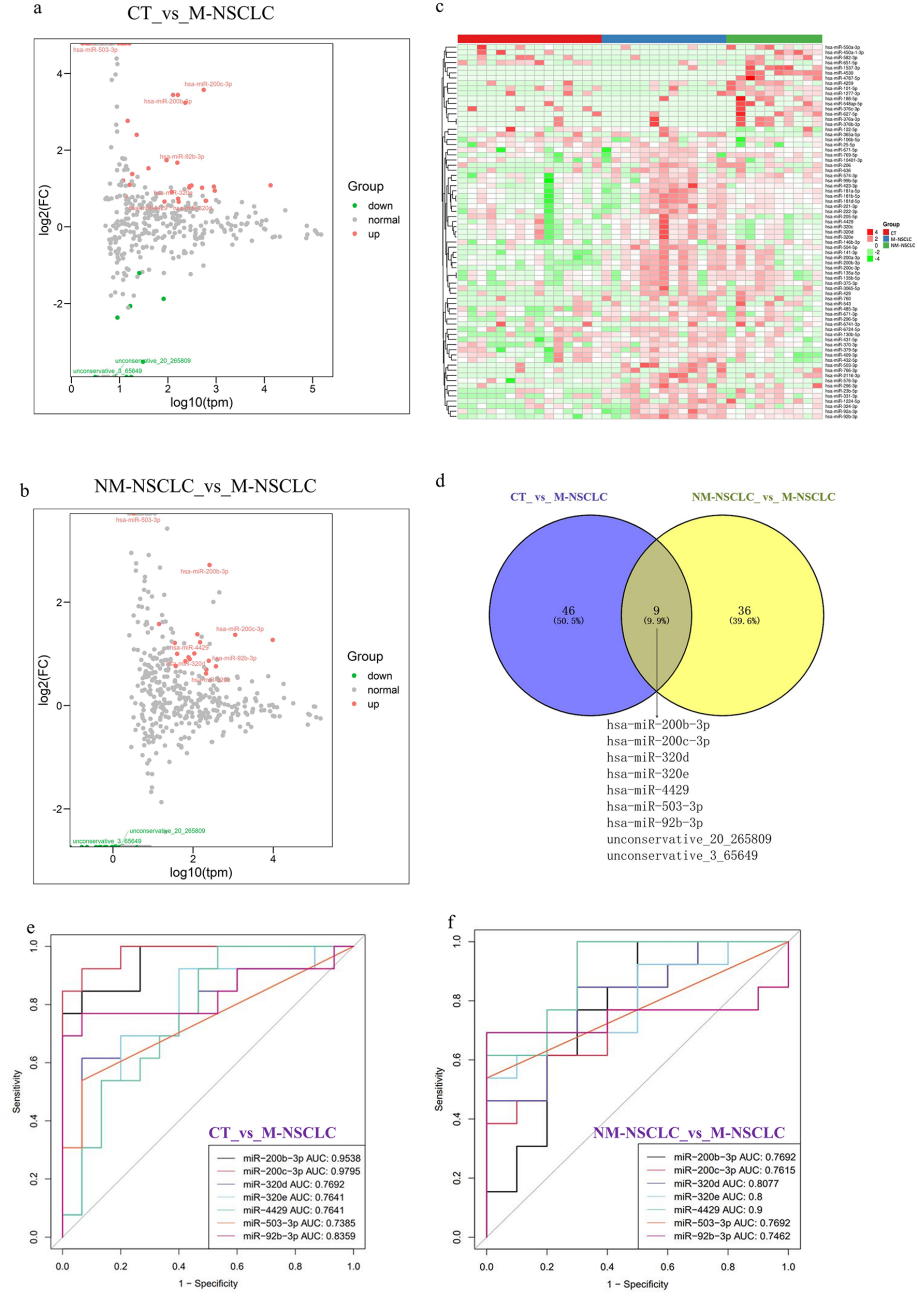
Identification and functional enrichment analysis of differentially expressed sEV-derived miRNAs. **a** & **b**. Volcano plot of differentially expressed sEV-derived miRNAs in the CT group versus M-NSCLC group and NM-NSCLC group versus M-NSCLC group. Each point represents a miRNA, red represents upregulated miRNA, green represents downregulated miRNA, and grey represents nondifferentially expressed miRNA. The marked miRNAs are the intersecting DEMs of the CT group versus the M-NSCLC group and the NM-NSCLC group versus the M-NSCLC group. **c**. The merged heatmap of DEMs of the CT group versus M-NSCLC group and NM-NSCLC group versus M-NSCLC group. Red indicates relatively high expression, and green indicates relatively low expression. **d**. Venn diagram comparing differentially expressed sEV-derived miRNAs, and each circle represents the number of differentially expressed sEV-derived miRNAs between two conditions. **e** & **f**. The AUC of the 7 DEMs for identifying the CT group versus the M-NSCLC group and the NM-NSCLC group versus the M-NSCLC group in the plasma-derived sEV-enriched fraction miRNA dataset, as shown in ROC curves

Next-generation sequencing (NGS) was performed on sEV-derived miRNAs from plasma. This revealed the presence of a total of 1745 miRNAs, of which 1363 are known. We included miRNAs with an average TPM > 10 for differential expression analysis to avoid bias induced by miRNAs with relatively low expression levels. For the M-NSCLC group compared with the CT group, a total of 55 DEMs had a > 1.5-fold change and *P* < 0.05, of which 45 were upregulated and 10 downregulated. In comparison between the M-NSCLC group and the NM-NSCLC group, a total of 45 DEMs were identified, of which 19 DEMs were upregulated and 26 downregulated. These data are depicted as volcano plots (MA plots) in Fig. 3a, b. The merged heatmap of DEMs for the CT group versus M-NSCLC group and NM-NSCLC group versus M-NSCLC group is shown in Fig. 3c.

Venn diagrams were plotted to identify miRNAs of plasma-derived sEVs that could be used for identification of metastatic NSCLC (Fig. 3d). Among the 9 miRNAs that were differentially expressed in the CT, NM-NSCLC, and M-NSCLC groups, 7 miRNAs (miRNA-200b-3p, miRNA-200c-3p, miRNA-320e, miRNA-320d, miRNA-4429, miRNA-503-3p and miRNA-92b-3p) are known and 2 (unconservative_20_265809 and unconservative_ 3_65649) unknown. The specificity and sensitivity of 7 DEMs for the CT group versus the M-NSCLC group and the NM-NSCLC group versus the M-NSCLC group using the plasma-derived sEV-enriched fraction miRNA dataset are shown by ROC curves in Fig. 3e, f. Expression levels of 7 miRNAs were significantly upregulated in the M-NSCLC group compared with the NM-NSCLC or CT group (Table S3).

### Functional annotation and identification of DEMs

**Fig. 4:**
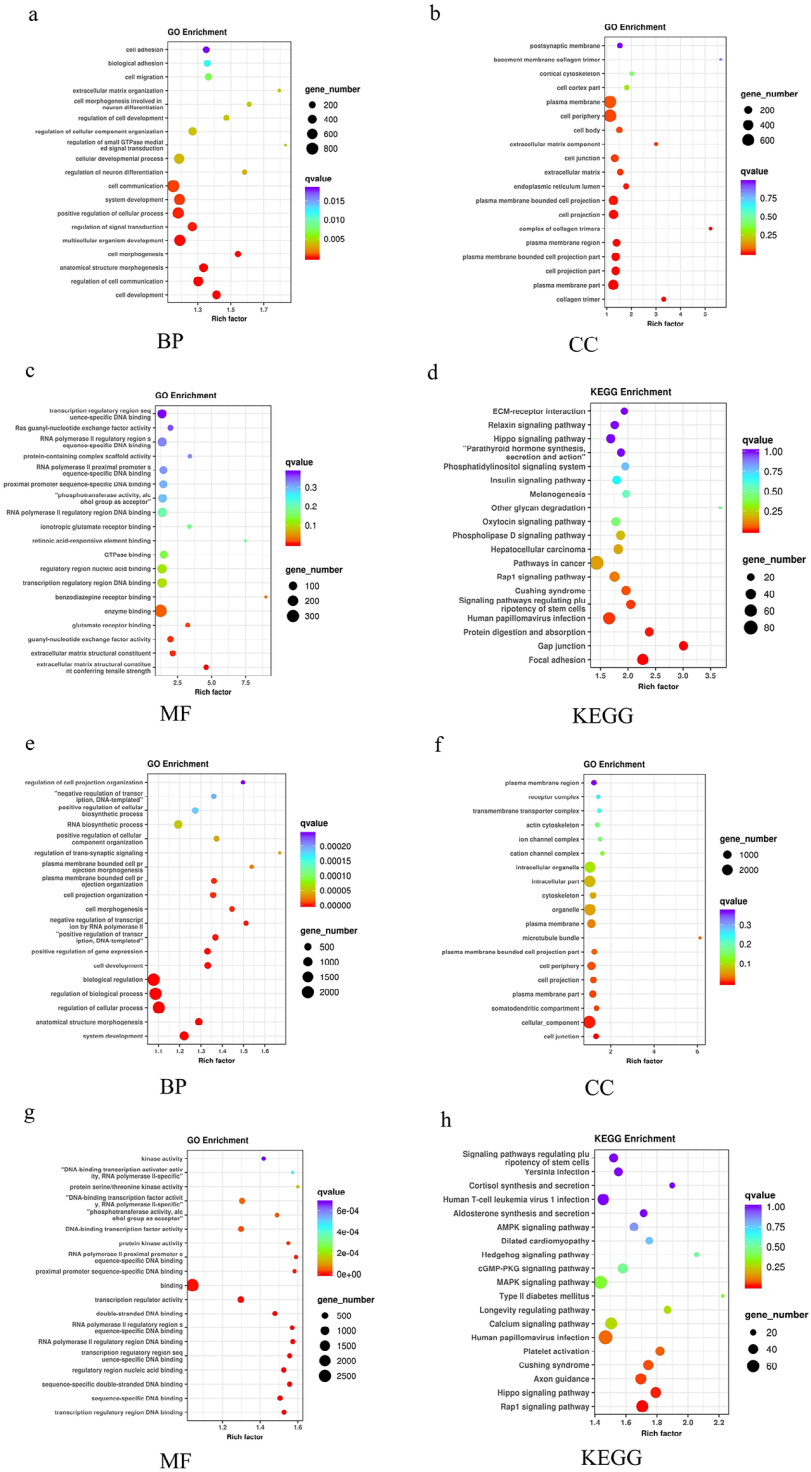
GO and KEGG pathway enrichment analyses of DEMs between the three groups. The advanced bubble chart shows enrichment of genes in signaling pathways. The x-axis label represents the enrichment factor, and the y-axis label represents the pathway. The colour and size of the bubbles represent the amount of DEMs enriched in each pathway and the enrichment significance, respectively. **a-c**. The GO enrichment for biological process, cellular component, and molecular function of DEMs between NM-NSCLC and M-NSCLC groups. **d**. The KEGG pathway of DEMs between NM-NSCLC and M-NSCLC groups. **e-g**. GO enrichment for biological process, cellular component, and molecular function of DEMs between the CT and M-NSCLC groups. **h**. The KEGG pathway of DEMs between the CT and M-NSCLC groups. Abbreviations: BP, biological process; CC, cellular component; MF, molecular function

GO and KEGG pathway enrichment analyses were conducted to understand the biological characteristics of DEMs. GO and KEGG pathway enrichment analyses were applied to analyse 45 DEMs in the NM-NSCLC group versus the M-NSCLC group. For biological processes, the most significantly enriched terms were in cell development, anatomical structure morphogenesis, and cell morphogenesis (P = 3.34E-08, 1.27E-06, and 1.10E-05, respectively). For cellular components, the most significantly enriched terms were plasma membrane part, extracellular matrix (ECM), and cell junction (P = 1.03E-04, 0.0185, and 0.0233, respectively). For molecular function, the most significantly enriched terms were ECM structural constituent, guanyl-nucleotide exchange factor activity, and enzyme binding (P = 0.0054, 0.0061, and 0.0202, respectively). The results of GO enrichment analysis are shown in Fig. 4a-c. KEGG pathway analysis revealed significant differences in pathways of focal adhesion, gap junctions, protein digestion and absorption, and signaling pathways regulating the pluripotency of stem cells (P = 9.00E-06, 1.42E-05, 0.0054, and 0.0202, respectively), as shown in Fig. 4d.

Furthermore, the biological characteristics of the 55 DEMs were compared between the M-NSCLC and CT groups. The GO terms are shown in Fig. 4e-g. For biological process, the DEMs were enriched in regulation of cellular process and plasma membrane-bounded cell projection organization (P = 2.06E-10 and 3.52E-06, respectively). For cellular component, the most significantly enriched terms were cell junction and plasma membrane-bounded cell projection part (P = 1.74E-04 and 0.0217). Regarding molecular function, the most significantly enriched terms were transcription regulator activity and protein serine/threonine kinase activity (P = 8.29E-07 and 1.36 E-04, respectively). KEGG pathway analysis showed that the Rap1 signaling pathway and Hippo signaling pathway were significantly different (P = 7.57 E-04 and 0.0157), as shown in Fig. 4h.

### ClueGO enrichment analysis

**Fig. 5:**
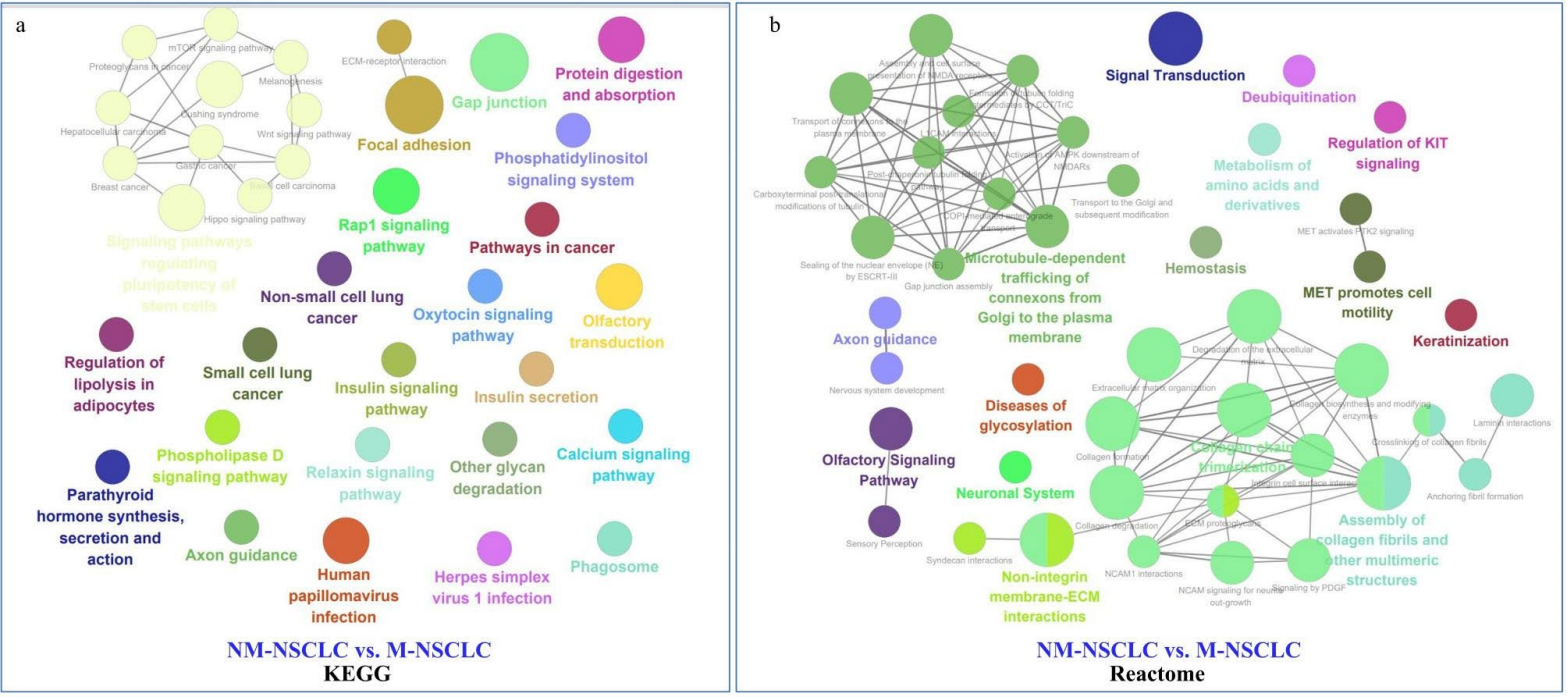
Functional enrichment analysis using ClueGO. (**a**) Functionally enriched KEGG pathways associated with DEMs between the NM-NSCLC group and the M-NSCLC group. Functionally grouped network with terms as nodes linked based on a kappa score of 0.5 was considered in the bilateral hypergeometric test. The color gradient shows the gene proportion of each cluster associated with the term. The node size represents the term enrichment significance. (**b**) Functionally enriched Reactome associated with DEMs between the NM-NSCLC group and the M-NSCLC group

The ClueGO/CluePedia plugin of Cytoscape software was employed to assess and examine the functional annotation of the identified DEGs. KEGG and Reactome pathways of DEMs were compared between the NM-NSCLC and M-NSCLC groups. KEGG pathways were significantly enriched in the Rap1 signaling pathway, Hippo signaling pathway, proteoglycans in the cancer mTOR signaling pathway, and Wnt signaling pathway (P < 0.045), which are shown in Fig. 5a. Reactome pathways were enriched in regulation of KIT signaling, and MET was shown to activate the PTK2 signaling pathway by PDGF, which is shown in Fig. 5b. The results of KEGG and Reactome pathway analyses are listed in Tables S4 and 5.

### MiRNA enrichment analysis and annotation tool analysis

miEAA was applied to compare enrichment of DEMs to understand changes in cell type and cell tissue origin from the occurrence of NSCLC to metastasis. The DEMs in the NM-NSCLC group versus M-NSCLC group were over-represented in fibroblasts (P = 0.0854), and were under-represented in CD3, CD19, and CD56 (P = 0.0247, 0.0833, and 0.0857, respectively). The results are shown in Table S6.

### qRT‒PCR validation and target gene prediction for miRNAs

**Fig. 6:**
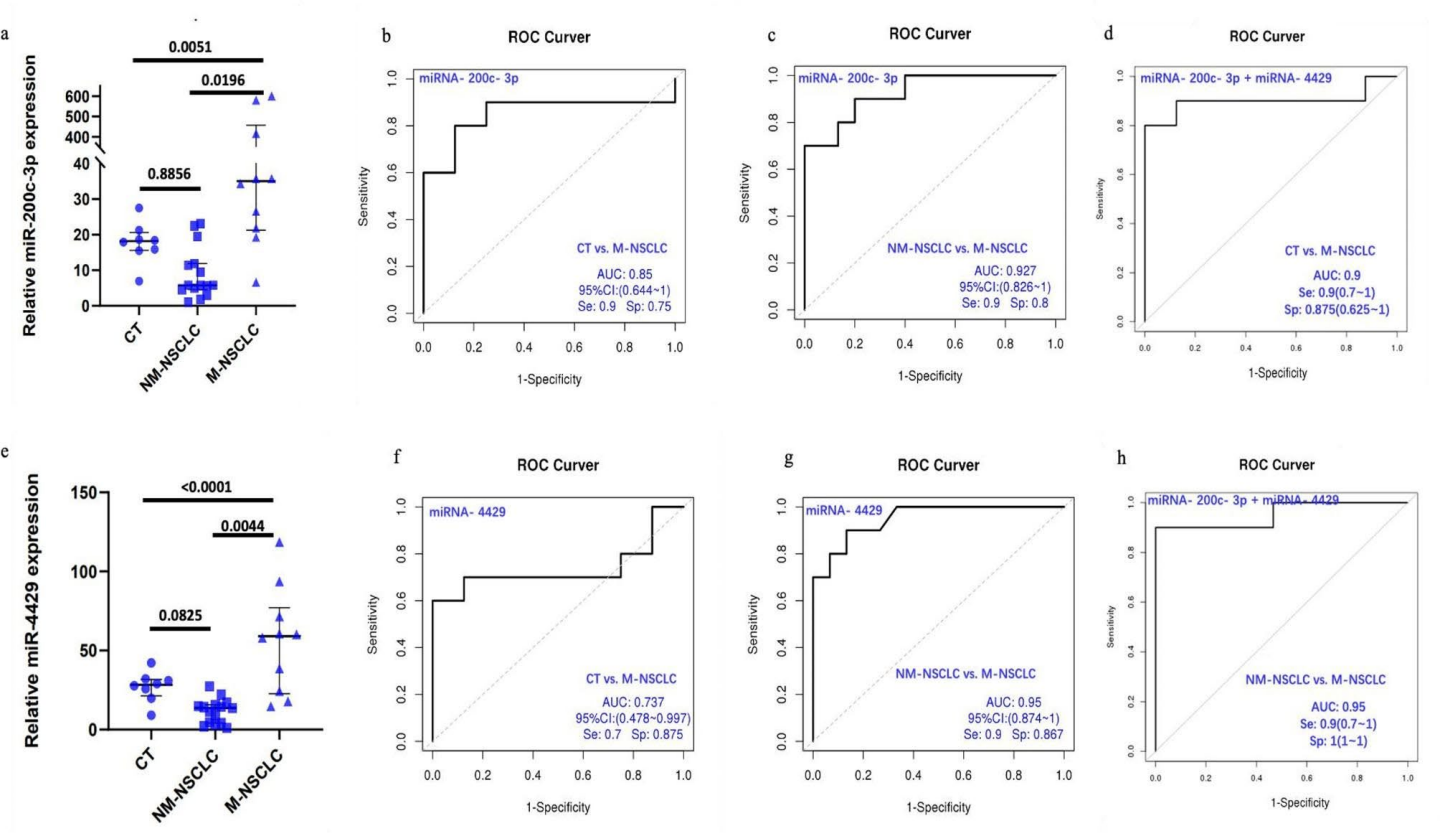
qPCR validation and ROC curves. **a** & **e**. Expression levels of miRNA-200c-3p and miRNA-4429 were higher in the CT group versus the M-NSCLC group and in the NM-NSCLC group versus the M-NSCLC group, respectively. The x-axis label represents the group, and the y-axis label represents the relative expression levels of miRNA. **b** & **c**. ROC curves of miRNA-200c-3p in the CT group versus the M-NSCLC group and in the NM-NSCLC group versus the M-NSCLC group. **f** & **g**. ROC curves of miRNA- 4429 in the CT group versus the M-NSCLC group and in the NM-NSCLC group versus the M-NSCLC group. **d** & **h**. Combined ROC curves of miRNA-200c-3p and miRNA-4429 in the CT group versus the M-NSCLC group and in the NM-NSCLC group versus the M-NSCLC group

In this study, 8 healthy controls were assigned to the CT group, 15 patients without distant organ metastasis were assigned to the NM-NSCLC group, and 10 patients with distant organ metastasis were assigned to the M-NSCLC group. The participants’ demographic and clinical data, such as age, sex, smoking history, tumour stage, pathological type, and metastatic site, are summarized in Table 1 and Table S1. Differential sEV-derived miRNAs from 33 participants were verified and compared among the CT, NM-NSCLC, and M-NSCLC groups. On the basis of the screening of expression levels and AUC results, miRNA-200c-3p and miRNA-4429 were selected as target miRNAs for further analysis; the primer sequences used for qRT‒PCR are shown in Table S1. Gene expression levels were calculated using the 2 ^−ΔΔCT^ method. The expression level of miRNA-200c-3p was significantly upregulated in the M-NSCLC group compared with the CT group and NM-NSCLC group (CT group vs. NM-NSCLC group, *P* = 0.8856; CT group vs. M-NSCLC group, *P* = 0.0196; NM-NSCLC group vs. M-NSCLC group, *P* = 0.0051) (Fig. 6a). The expression level of miRNA- 4429 was significantly upregulated in the M-NSCLC group compared with the CT group and NM-NSCLC group (CT group vs. NM-NSCLC group, *P* = 0.0825; CT group vs. M-NSCLC group, *P* = 0.0044; NM-NSCLC group vs. M-NSCLC group, *P* < 0.0001) (Fig. 6e). Furthermore, ROC curve analysis was performed to calculate the diagnostic value of the two miRNAs. ROC curves of miRNA-200c-3p and miRNA-4429 were compared between the M-NSCLC and CT groups, and higher values of specificity and sensitivity were obtained (miRNA-200c-3p: AUC = 0.85, specificity = 0.9, sensitivity = 0.75; miRNA-4429: AUC = 0.737, specificity = 0.875, sensitivity = 0.7) (Fig. 6b, f). ROC curves of miRNA- 200c-3p and miRNA-4429 were compared between the M-NSCLC and NM-NSCLC groups, and higher values of specificity and sensitivity were obtained (miRNA-200c-3p: AUC = 0.927, specificity = 0.8, sensitivity = 0.9; miRNA-4429: AUC = 0.95, specificity = 0.867, sensitivity = 0.9) (Fig. 6c, g). In addition, combined analysis of miRNA-200c-3p and miRNA-4429 resulted in higher values of specificity and sensitivity compared with M-NSCLC versus CT and M-NSCLC versus NM-NSCLC (AUC = 0.927, specificity = 0.9, sensitivity = 0.875; AUC = 0.95, specificity = 0.9, sensitivity = 1) (Fig. 6d, h).

### Prognostic risk of miRNA-200c-3p and miRNA-4429 in NSCLC based on TCGA data

**Fig. 7:**
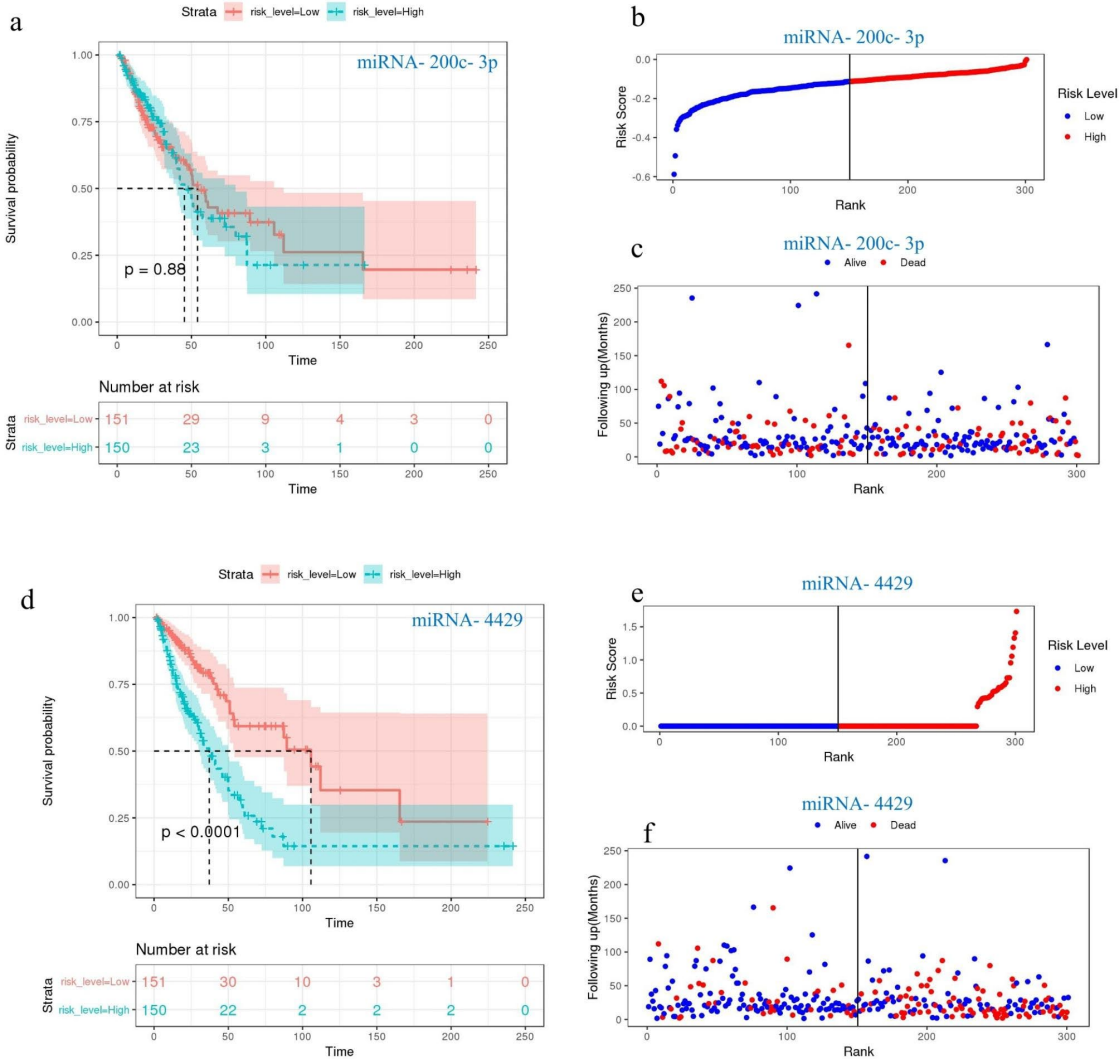
Prognostic risk of miRNA-200c-3p and miRNA-4429 in NSCLC from TCGA data. **a** & **d**. Kaplan‒Meier curve for the risk score model in the miRNA-200c-3p and miRNA-4429 datasets. **b** & **e**. The distribution of risk scores. The black dotted line represents the median risk score after dividing patients into low-risk and high-risk groups. **c** & **f**. Scatter diagram of the expression distribution of alive or dead in the model sorted by risk score

MiRNA-seq and survival data of NSCLC patients were downloaded from TCGA to further validate the effects of miRNA-200c-3p and miRNA-4429 on NSCLC development. In total, 301 patients were divided into two groups on the basis of the median value of the risk score used for predicting miRNA expression levels in NSCLC patients (150 high-risk patients and 151 low-risk patients). The survival curve revealed that the miRNA-200c-3p expression level was not associated with a significantly different OS between the high-risk group and the low-risk group (P = 0.88), as shown in Fig. 7a-c. However, upregulated miRNA-4429 in the high-risk group was significantly associated with poorer OS than that in the low-risk group (P = 2.29E-06), as illustrated in Fig. 7d-f.

### Target genes of the two miRNAs

**Fig. 8:**
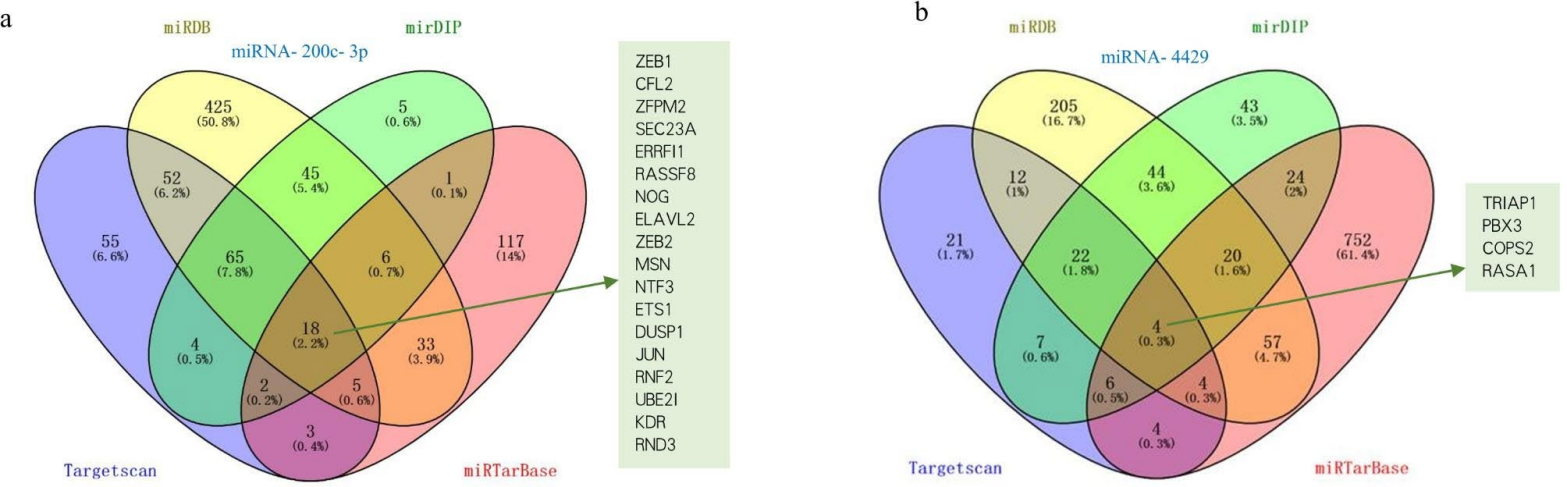
Predicted target genes were intersecting genes downloaded from the TargetScan, miRDB, mirDIP, and miRTarBase databases. (**a**) Predicted target genes of miRNA-200c-3p. (**b**) Predicted target genes of miRNA-4429. Venn diagram represents the common target genes of miRNA-200c-3p or miRNA-4429 in the four websites

MiRNA-200c-3p and miRNA-4429 data were downloaded from the TargetScan, miRDB, mirDIP, and miRTarBase databases. The intersecting genes of the four websites were selected as target genes, which are shown as Venn diagrams in Fig. 8a, b. Moreover, ZEB1, CFL2, ZFPM2, RASSF8, ZEB2, and other genes were selected as target genes for miRNA-200c-3p or miRNA-4429, and the pathways and references are summarized in Table S7.

## Discussion

Metastasis, a process of cancer cells spreading from the primary tumour to distant organs, is the primary cause of cancer mortality. It is estimated that metastasis is responsible for 90% of cancer-related deaths [25]. Lung cancer is the leading cause of cancer-related death worldwide and has a propensity for distant metastasis. A better understanding of the molecular events that regulate the development of metastatic lung cancer and biomarkers that predict which patients may experience metastasis is suggested to improve survival rates [26]. In recent years, multiple studies have shown that sEVs are abundantly secreted into the circulation by several cells and contain proteins from the plasma and endosomal membranes, as well as proteins, RNAs, and DNAs that reflect the phenotypic state of their cells of origin [27]. Furthermore, DEMs of sEVs derived from plasma have been reported to play an essential role in progression of various types of cancer, including NSCLC, by regulating expression levels of multiple target genes involved in progression and metastasis [28, 29]. Thus, identification of specific miRNAs of plasma-sEVs and their targets involved in carcinogenesis would provide valuable insights into the diagnosis and therapy of patients with malignancies.

In the present study, miRNAs from plasma-sEVs of 38 samples were isolated, including healthy controls, NM-NSCLC and M-NSCLC patients, and differences in miRNAs were compared among the three groups. By GO and KEGG pathway enrichment analyses, we found that for biological process, M-NSCLC showed enrichment in cell junction, regulation of signal transduction, and multicellular organism development, with the Rap1 and Hippo pathways being enriched, confirming that the DEMs from plasma sEVs are involved in metastatic NSCLC. Furthermore, we analysed DEMs between NM-NSCLC and M-NSCLC patients through ClueGO enrichment analyses and found that the Rap1 and Hippo signaling pathways are important for metastatic NSCLC. Several studies have confirmed that the Rap1 and Hippo signaling pathways play a key role in tumour cell proliferation and apoptosis, which might be related to tumour growth [30, 31]. Rap1 is a small GTPase belonging to the Ras family, marking the malignant expression of fibroblasts transformed by KRAS [32]. The Ras family regulates the biological behaviours of various tumours, such as cytoadhesion, growth, migration, and gene mutations, causing the occurrence of malignant tumours [33]. A high expression level of Rap1 can impair cell adhesion or enhance cell-matrix adhesion, which can promote tumour cell migration and invasion [34]. The Hippo signaling pathway, initially identified by a Drosophila mosaic screen for an overgrowth phenotype, has been proven to play an important role in different types of human cancer, acting as either a tumour suppressor or a tumour promoter [35–37]. In our study, we predicted that the Rap1 and Hippo signaling pathways are of great importance to metastatic NSCLC, and the results provide a theoretical basis for further research on sEV-derived miRNAs from plasma samples and associated pathways.

It is generally accepted that progression and metastasis of cancer are controlled by the TME, not only by autonomous defects in cancer cells [38]. The tumour stroma is composed of immune cells, the basement membrane, capillaries, activated fibroblasts and the extracellular matrix (ECM) around cancer cells [39]. sEVs can be mediators of intercellular communication. Thus, we used miRNAs to determine the TME of M-NSCLC patients. As immune cells, the DEMs of NM-NSCLC versus M-NSCLC were enriched in CD3, CD19, and CD56 cells for under-represented. The CD3 component of the T-cell receptor complex CD19 is an antigen marker of B cells, and most CD5 antigens are expressed in natural killer (NK) cells [40–42]. These results demonstrate that immune cells are involved in metastatic NSCLC. Immune checkpoint inhibitors are widely used in the clinic, and they have revolutionized treatment of different types of cancer [43]; however, the biomarkers for immune checkpoint inhibitors are unclear, and CD3, CD19, and CD56 cells will become new immunotherapeutic targets.

Fibroblasts are a dominant component of the tumour stroma, and several studies have suggested a prominent functional role of these cells in cancer progression and metastasis [44, 45]. Fibroblasts associated with cancer have been termed cancer-associated fibroblasts (CAFs), tumour-associated fibroblasts, activated fibroblasts or activated myofibroblasts, which may include cancer-related mesenchymal stem cells. CAFs are one of the most abundant stromal cells in the TME [46], and they can establish and reshape the structure of the ECM, facilitate tumour cells invading blood vessels, interact with tumour cells or other stromal cells, and promote tumour progression by secreting cytokines, growth factors and chemokines [47, 48]. Due to the high heterogeneity of CAFs and the lack of specific markers for their origin, classification and biological function, the role of CAFs has not been comprehensively evaluated, and there are still some disputes. In the study of miRNAs, DEMs were enriched in fibroblasts and had high activities in metastatic NSCLC patients, which indicates that fibroblasts may play an important role in metastatic NSCLC and TME changes at different tumour stages.

It is well known that the miR-200 family (miR-200a, miR-200b, miR-200c, miR-429, and miR-141) plays an important role in the epithelial–mesenchymal transition (EMT), which is important for tumour metastasis [49, 50]. Numerous studies on NSCLC have provided evidence for a critical role of miR-200c in regulating the EMT, ubiquitin-specific peptidase 25 (USP25), cathepsin L (CTSL), and hypoxia-inducible factor-1α (HIF-1α), which are prerequisites for the formation of metastases and the primary cause of cancer mortality [51–53]. In this study, the expression level of miRNA-200c-3p derived from sEVs of plasma samples was significantly upregulated in M-NSCLC patients, accompanied by higher sensitivity and specificity. It was previously reported that miRNA-4429 was detected in the plasma of patients with acute ischaemic stroke, and it was found that the expression level of miRNA-4429 was significantly reduced in acute ischaemic stroke patients [54]. Studies confirmed that miR-4429 is dysregulated in cancer cells, and it was demonstrated to play an important role in diverse types of cancer. For instance, the role of miR-4429 includes targeting METTL3 to inhibit m6A-induced stabilization, targeting RAD51, targeting distal-less homeobox 1, and inactivating the Wnt/β-catenin pathway [55–57]. In this study, the expression level of miRNA-4429 derived from plasma-sEVs was significantly upregulated in M-NSCLC patients, accompanied by higher specificity and sensitivity.

In addition, miRNA-seq and survival data for miRNA-200c-3p and miRNA- 4429 of NSCLC patients were downloaded from TCGA, and miRNA-4429 correlated with the prognosis of NSCLC patients, though this finding was not confirmed for miRNA-200c-3p. However, the data obtained from TCGA are expression levels in NSCLC patient tissues, which could be different from the expression data of miRNA in plasma-sEVs. Although our results were slightly different from those of TCGA, miRNAs of plasma-sEVs can well reflect the TME state. Hence, it was confirmed that these two genes can be used as prognostic genes for NSCLC patients, especially miRNA-4429.

TargetScan, miRDB, mirDIP, and miRTarBase are common gene databases, and we used them to identify target genes for miRNA-200c-3p and miRNA-4429. Through analysis, it was confirmed that ZEB1, JUN, ETS1, ERRFI1, and DUSP1 are target genes of miRNA-200c-3p; the target genes of miRNA-4429 are predicted to be COPS2, PBX3, RASA1, and TRIAP1. These genes promote tumour growth and metastasis by acting on multiple pathways and were reported in tumour-related studies [58–68]. We found that several studies on ZEB1 have been conducted but that few studies have focused on CFL2, ELAVL2, and MSN. Therefore, prediction of these target genes indicates the direction for our future research.

This study has several limitations. First, the number of participating patients was small. If the sample size can be expanded, more reliable conclusions may be drawn. However, several patients in our department had already been treated in other hospitals, and the number of patients without uncomplications treated for the first time was also limited. Second, this was an exploratory study on the mechanism of tumour metastasis, and the patients benefited less from short-term therapy, leading to the participation of fewer patients. Third, we predicted target genes, and further evidence is required to confirm them. However, this research is very meaningful, and the results provide a promising theoretical basis for the mechanism of metastatic NSCLC. Further study is essential to verify our predicted target genes.

## Conclusions

In summary, this study confirmed that miRNA-200c-3p and miRNA-4429 derived from plasma-sEVs can be used as potential biomarkers for metastatic NSCLC and predict the potential target pathway for metastatic NSCLC.

## Electronic supplementary material

Below is the link to the electronic supplementary material.

**Supplementary materials**: **Table S1**. Primer sequences for qRT- PCR. **Table S2**. The clinical characteristics of NM-NSCLC group and M-NSCLC group patients. **Table S3**. The expression levels of seven miRNAs are significantly upregulated in group CT versus M-NSCLC group and NM-NSCLC group versus M-NSCLC group. **Table S4**. ClueGO enrichment analysis of KEGG result for DEMs between NM-NSCLC group and M-NSCLC group. **Table S5**. ClueGO enrichment analysis of reactome result for DEMs between NM-NSCLC group and M-NSCLC group. **Table S6**. The miEAA results of DEMs between NM-NSCLC group and M-NSCLC group. **Table S7**. The reference of miRNA- 200c- 3p and miRNA- 4429 for predicted target genes’ functions and pathways in NSCLC progression and metastasis.

## Data Availability

The datasets generated and analysed during the current study are available in the China National Center for Bioinformation/Beijing Institute of Genomics, Chinese Academy of Sciences (https://ngdc.cncb.ac.cn/omix: accession No. OMIX002481 or https://ngdc.cncb.ac.cn/omix/release/OMIX002481).

## List of abbreviations

miRNAs: microRNAs
sEVs: Small extracellular vesicles
NSCLC: Non-small cell lung cancer
CT: Control
NGS: Next-generation sequencing
DEMs: Differentially expressed miRNAs
KEGG: Kyoto Encyclopedia of Genes and Genomes
miEAA: miRNA enrichment analysis and annotation tool
qRT‒PCR: Quantitative reverse transcription polymerase chain reaction
3′-UTR: 3′-untranslated region
EVs: Extracellular vesicles
MVs: Microvesicles
mRNAs: Messenger RNAs
TME: Tumour microenvironment
miRNA-seq: miRNA sequencing
DEMs: Differentially expressed miRNAs
GO: Gene Ontology
TCGA: The Cancer Genome Atlas
SEC: Size exclusion chromatography
PBS: Phosphate-buffered saline
NTA: Nanoparticle tracking analysis
TEM: Transmission electron microscopy
SDS: Sodium dodecyl sulfonate
rRNA: Ribosomal RNA
tRNA: Transfer RNA
snRNA: Small nuclear RNA
snoRNA: Small nucleolar RNA
ORA: Overrepresentation analysis
ROC: Receiver operating characteristic
AUC: Area under the ROC curve
ANOVA: Analysis of variance
EMT: Epithelial–mesenchymal transition
USP25: Ubiquitin-specific peptidase 25
CTSL: Cathepsin L
HIF-1α: Hypoxia-inducible factor-1α
