# Supplementary material for: Two microRNAs of plasma-derived small extracellular vesicles as biomarkers for metastatic non-small cell lung cancer
Source: BMC Pulm Med. 2023 Jul 14;23:259. doi: 10.1186/s12890-023-02538-w (PMC10347730; doi:10.1186/s12890-023-02538-w)
Supplement: Supplementary file 1 — Supplementary materials: Table S1. Primer sequences for qRT- PCR. Table S2. The clinical characteristics of NM-NSCLC group and M-NSCLC group patients. Table S3. The expression levels of seven miRNAs are significantly upregulated in group CT versus M-NSCLC group and NM-NSCLC group versus M-NSCLC group. Table S4. ClueGO enrichment analysis of KEGG result for DEMs between NM-NSCLC group and M-NSCLC group. Table S5. ClueGO enrichment analysis of reactome result for DEMs between NM-NSCLC group and M-NSCLC group. Table S6. The miEAA results of DEMs between NM-NSCLC group and M-NSCLC group. Table S7. The reference of miRNA- 200c- 3p and miRNA- 4429 for predicted target genes’ functions and pathways in NSCLC progression and metastasis. [file 12890_2023_2538_MOESM1_ESM.docx]

**Supplementary materials**

**Table S1. Primer sequences for qRT- PCR.**

| Gene ID | Primer sequences | | |
| --- | --- | --- | --- |
|  | Forward(5'--3') | Reverse(5'--3') | Probe |
| miRNA- 200c- 3p | ACGCTAATACTGCCGGGTAAT | GTCGTATCCAGTGCAGGGTCCGAGGTATTCGCACTGGATACGACTCCATC | TTCGCACTGGATACGACTCCATC |
| miRNA- 4429 | CACGAAAAGCTGGGCTGA | GTCGTATCCAGTGCAGGGTCCGAGGTATTCGCACTGGATACGACCGCCTC | TTCGCACTGGATACGACCGCCTC |
| cel- miRNA- 39 | CGCTCACCGGGTGTAAATC | GTCGTATCCAGTGCAGGGTCCGAGGTATTCGCACTGGATACGACCAAGCT | ATTCGCACTGGATACGACCAAGCT |

**Table S2. The clinical characteristics of NM-NSCLC group and M-NSCLC group patients**

| **Group** | **Identifier** | **Sex** | **Age**  **(year)** | **Smoking history** | **Pathological type** | **Clinical stages** | **Metastatic organs** |
| --- | --- | --- | --- | --- | --- | --- | --- |
| NM-NSCLC  (Screening stage) | 1 | Female | 62 | No | Adenocarcinoma | II | - |
|  | 2 | Female | 67 | No | Adenocarcinoma | II | - |
|  | 3 | Male | 68 | Yes | Adenocarcinoma | II | - |
|  | 4 | Female | 60 | No | Adenocarcinoma | II | - |
|  | 5 | Male | 71 | No | Adenocarcinoma | I | - |
|  | 6 | Male | 79 | No | Adenocarcinoma | II | - |
|  | 7 | Female | 34 | No | Adenocarcinoma | II | - |
|  | 8 | Female | 61 | Yes | Squamous cell carcinoma | I | - |
|  | 9 | Male | 57 | Yes | Squamous cell carcinoma | II | - |
|  | 10 | Male | 67 | Yes | Squamous cell carcinoma | II | - |
| NM-NSCLC  (validation stage) | 1 | Male | 59 | No | Adenocarcinoma | II | - |
|  | 2 | Male | 68 | No | Adenocarcinoma | II | - |
|  | 3 | Female | 57 | No | Adenocarcinoma | II | - |
|  | 4 | Male | 70 | Yes | Squamous cell carcinoma | I | - |
|  | 5 | Male | 61 | No | Squamous cell carcinoma | II | - |
|  | 6 | Male | 70 | Yes | Squamous cell carcinoma | II | - |
|  | 7 | Female | 57 | No | Squamous cell carcinoma | I | - |
|  | 8 | Male | 66 | No | Squamous cell carcinoma | II | - |
|  | 9 | Female | 66 | No | Squamous cell carcinoma | II | - |
|  | 10 | Female | 72 | No | Squamous cell carcinoma | II | - |
|  | 11 | Male | 64 | No | Squamous cell carcinoma | II | - |
|  | 12 | Male | 58 | Yes | Squamous cell carcinoma | II | - |
|  | 13 | Male | 59 | Yes | Squamous cell carcinoma | II | - |
|  | 14 | Male | 72 | Yes | Squamous cell carcinoma | II | - |
|  | 15 | Male | 74 | Yes | Squamous cell carcinoma | II | - |
| M-NSCLC  (Screening stage) | 1 | Female | 65 | No | Adenocarcinoma | IV | Brain |
|  | 2 | Male | 67 | Yes | Adenocarcinoma | IV | Boan |
|  | 3 | Female | 72 | No | Adenocarcinoma | IV | Boan |
|  | 4 | Male | 53 | Yes | Squamous cell carcinoma | IV | Boan |
|  | 5 | Male | 54 | No | Adenocarcinoma | IV | Pleura |
|  | 6 | Male | 69 | Yes | Adenocarcinoma | IV | Brain |
|  | 7 | Female | 52 | No | Adenocarcinoma | IV | Pleura |
|  | 8 | Female | 62 | No | Adenocarcinoma | IV | Brain |
|  | 9 | Female | 56 | No | Adenocarcinoma | IV | Brain |
|  | 10 | Female | 62 | Yes | Adenocarcinoma | IV | Brain |
|  | 11 | Male | 51 | No | Adenocarcinoma | IV | Brain |
|  | 12 | Male | 66 | No | Adenocarcinoma | IV | Boan |
|  | 13 | Male | 67 | No | Adenocarcinoma | IV | Adrenal gland |
| M-NSCLC  (validation stage) | 1 | Male | 64 | Yes | Squamous cell carcinoma | IV | Boan |
|  | 2 | Female | 68 | No | Squamous cell carcinoma | IV | Liver |
|  | 3 | Male | 72 | Yes | Squamous cell carcinoma | IV | Brain |
|  | 4 | Male | 60 | No | Adenocarcinoma | IV | Brain |
|  | 5 | Male | 31 | No | Adenocarcinoma | IV | Brain |
|  | 6 | Male | 33 | Yes | Squamous cell carcinoma | IV | Boan |
|  | 7 | Male | 64 | Yes | Adenocarcinoma | IV | Adrenal gland |
|  | 8 | Male | 72 | No | Adenocarcinoma | IV | Liver |
|  | 9 | Male | 55 | No | Adenocarcinoma | IV | Brain |
|  | 10 | Female | 45 | No | Adenocarcinoma | IV | Brain |

**Table S3. The expression levels of seven miRNAs are significantly upregulated in** **group CT versus M-NSCLC group and NM-NSCLC group versus M-NSCLC group.**

| MiRNA | CT versus M-NSCLC | | | NM-NSCLC versus M-NSCLC | | |
| --- | --- | --- | --- | --- | --- | --- |
|  | PValue | log2FC | regulated | PValue | log2FC | regulated |
| hsa-miR-200b-3p | 4.63506E-05 | 3.440994663 | up | 0.03032867 | 2.721174196 | up |
| hsa-miR-200c-3p | 6.40989E-07 | 3.571878026 | up | 0.035844086 | 1.368248596 | up |
| hsa-miR-320d | 0.014835255 | 0.657062534 | up | 0.012095456 | 0.696908238 | up |
| hsa-miR-320e | 0.01702749 | 0.735155707 | up | 0.01471069 | 0.621004192 | up |
| hsa-miR-4429 | 0.01702749 | 0.658893961 | up | 0.000639823 | 1.006911521 | up |
| hsa-miR-503-3p | 0.007882971 | Inf | up | 0.008648184 | Inf | up |
| hsa-miR-92a-3p | 0.028717574 | 1.084320795 | up | 0.049329322 | 0.867168314 | up |

**Table S4. ClueGO enrichment analysis of KEGG result for DEMs between NM-NSCLC group and M-NSCLC group.**

| Group comparison | ID | Term | Term P value | Term P value corrected with Benjamini-Hochberg |
| --- | --- | --- | --- | --- |
| NM-NSCLC versus M-NSCLC | KEGG:00511 | Other glycan degradation | 0.00 | 0.02 |
|  | KEGG:04015 | Rap1 signaling pathway | 0.00 | 0.00 |
|  | KEGG:04926 | Relaxin signaling pathway | 0.00 | 0.03 |
|  | KEGG:04974 | Protein digestion and absorption | 0.00 | 0.00 |
|  | KEGG:05165 | Human papillomavirus infection | 0.00 | 0.00 |
|  | KEGG:05168 | Herpes simplex virus 1 infection | 0.00 | 0.01 |
|  | KEGG:05200 | Pathways in cancer | 0.00 | 0.01 |
|  | KEGG:04070 | Phosphatidylinositol signaling system | 0.00 | 0.02 |
|  | KEGG:05223 | Non-small cell lung cancer | 0.01 | 0.04 |
|  | KEGG:04072 | Phospholipase D signaling pathway | 0.00 | 0.01 |
|  | KEGG:04145 | Phagosome | 0.01 | 0.04 |
|  | KEGG:04540 | Gap junction | 0.00 | 0.00 |
|  | KEGG:04923 | Regulation of lipolysis in adipocytes | 0.01 | 0.04 |
|  | KEGG:04020 | Calcium signaling pathway | 0.00 | 0.04 |
|  | KEGG:04510 | Focal adhesion | 0.00 | 0.00 |
|  | KEGG:04512 | ECM-receptor interaction | 0.00 | 0.04 |
|  | KEGG:04150 | mTOR signaling pathway | 0.00 | 0.04 |
|  | KEGG:04310 | Wnt signaling pathway | 0.00 | 0.04 |
|  | KEGG:04390 | Hippo signaling pathway | 0.01 | 0.04 |
|  | KEGG:04550 | Signaling pathways regulating pluripotency of stem cells | 0.00 | 0.00 |
|  | KEGG:04934 | Cushing syndrome | 0.00 | 0.00 |
|  | KEGG:05205 | Proteoglycans in cancer | 0.01 | 0.04 |

**Table S5. ClueGO enrichment analysis of reactome result for DEMs between NM-NSCLC group and M-NSCLC group.**

| Group comparison | ID | Term | Term P value | Term P Value corrected with Benjamini-Hochberg |
| --- | --- | --- | --- | --- |
| NM-NSCLC versus M-NSCLC | R-HSA:71291 | Metabolism of amino acids and derivatives | 0.00 | 0.02 |
|  | R-HSA:162582 | Signal Transduction | 0.00 | 0.00 |
|  | R-HSA:1433559 | Regulation of KIT signaling | 0.00 | 0.01 |
|  | R-HSA:8874081 | MET activates PTK2 signaling | 0.00 | 0.04 |
|  | R-HSA:8875878 | MET promotes cell motility | 0.00 | 0.04 |
|  | R-HSA:3000171 | Non-integrin membrane- ECM interactions | 0.00 | 0.00 |
|  | R-HSA:3000178 | ECM proteoglycans | 0.00 | 0.04 |
|  | R-HSA:2022090 | Assembly of collagen fibrils and other multimeric structures | 0.00 | 0.00 |
|  | R-HSA:373760 | L1CAM interactions | 0.00 | 0.01 |
|  | R-HSA:389960 | Formation of tubulin folding intermediates by CCT/TriC | 0.00 | 0.01 |
|  | R-HSA:389977 | Post-chaperonin tubulin folding pathway | 0.00 | 0.02 |
|  | R-HSA:6807878 | COPI-mediated anterograde transport | 0.00 | 0.04 |
|  | R-HSA:8955332 | Carboxyterminal post-translational modifications of tubulin | 0.00 | 0.04 |
|  | R-HSA:948021 | Transport to the Golgi and subsequent modification | 0.00 | 0.04 |
|  | R-HSA:9609736 | Assembly and cell surface presentation of NMDA receptors | 0.00 | 0.00 |
|  | R-HSA:9619483 | Activation of AMPK downstream of NMDARs | 0.00 | 0.01 |
|  | R-HSA:9668328 | Sealing of the nuclear envelope (NE) by ESCRT-III | 0.00 | 0.00 |
|  | R-HSA:1442490 | Collagen degradation | 0.00 | 0.00 |
|  | R-HSA:1474228 | Degradation of the extracellular matrix | 0.00 | 0.00 |
|  | R-HSA:186797 | Signaling by PDGF | 0.00 | 0.00 |

**Table S6. The miEAA results of DEMs between NM-NSCLC group and M-NSCLC group.**

| Category | Subcategory | NM- NSCLC versus M- NSCLC | |
| --- | --- | --- | --- |
|  |  | Enrichment | P-value |
| Cell-type specific (Atlas) | Fibroblast | over-represented | 0.0854 |
| Immune cells | CD19 expressed | under-represented | 0.0833 |
|  | CD3 expressed | under-represented | 0.0247 |
|  | CD56 expressed | under-represented | 0.0857 |

**Table S7. The reference of miRNA- 200c- 3p and miRNA- 4429 for predicted target genes’ functions and pathways in NSCLC progression and metastasis.**

| miRNA | Target genes | Material | Function | Pathway | Cancer | Reference |
| --- | --- | --- | --- | --- | --- | --- |
| miRNA-200c-3p | DUSP1 | H460 cells | Invasion and metastasis | DUSP1/MKP1 | NSCLC | [58] |
|  | ETS1 | Tumor tissue/H522 and H460 cells | Progression | LINC01426/miR-519d-5p/ETS1 Axes | NSCLC | [59] |
|  | JUN | PC9 and HCC827 cells | Growth and metastasis | JNK–c-Jun pathway | NSCLC | [60] |
|  | KDR | Tumor tissues | Progression | VEGF/KDR, Tumor microvessel density increased | NSCLC | [61] |
|  | RASSF8 | H23, H1755, and H522 cells | Epithelial-mesenchymal transitioning and metastasis | circPTPRA/miR-96-5p/RASSF8/E-cadherin axis | NSCLC | [62] |
|  | RND3 | H358, H520 and A549 cells | Proliferation | Notch1/NICD/Hes1 signaling axis | NSCLC | [63] |
|  | ZEB1 | NCI-H1299, NCI-H1792, NCI-H2087, Calu-1, cells | Progresss | epithelial-to-mesenchymal transition | NSCLC | [64] |
|  | ZEB2 | A549, H1299, PC9 and SPCA-1/ tumor tissues | Metastasis | Slug/ZEB2 signaling | NSCLC | [65] |
|  | ZFPM2 | SPC-A1, A549, H1299,  and PC9 | Progression | ZFPM2-AS1–UPF1–ZFPM2 axis | NSCLC | [66] |
| miRNA-4429 | PBX3 | Tumor tissues / A549 cells/ Female nude mice | Progression | circNBPF10/miR-224 Axis | NSCLC | [67] |
|  | TRIAP1 | A549 and H460 cells | Increased apoptosis and intracellular reactive oxygen | TP53-regulated inhibitor of apoptosis 1 (TRIAP1) | NSCLC | [68] |

**Western blot original images：**

The 1, 2 well plate lanes were the WB result we submitted in the manuscript, 1 well plate lane was control and 2 well plate lane was sEVs. The control used A549 cell lysates (Procell Life Science&Technology Co.Ltd.，CL-0016). These images are shown in the example figure:


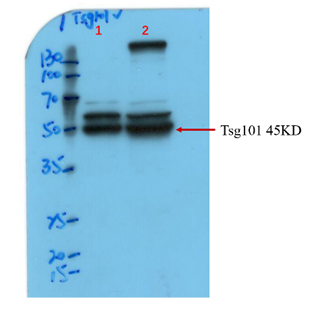

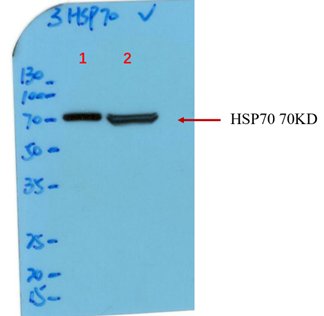

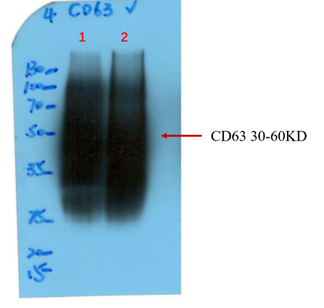

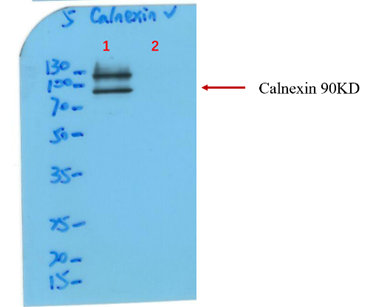


WB experiment process:

1) According to the molecular weight of the target protein, 12% and 8% separation gel were prepared, and the concentration of concentrated gel was 5%.

2) Loading amount of protein sample to be tested: 10μg- 30 μg / well.

3) Electrophoresis conditions: concentrated gel constant pressure 90V, about 20 minutes; The separation gel was kept at a constant pressure of 160V, and the electrophoresis stop time was determined by marker of pre stained protein.

4) Wet rotation method, film rotation conditions: 300 mA constant current; 0.45μm pore size NC membrane, film rotation time for 2 hours. After the completion of membrane transfer, Ponceau S was used to dye the membrane, observe the effect of membrane transfer, and mark the lane at the same time.

5) Blocking: the membrane was completely immersed in 3% BSA- TBST and gently shaken at room temperature for 30 minutes.

6) Primary antibody incubation: the primary antibody was diluted with 3% BSA- TBST, incubated at room temperature for 10min, and placed at 4 °C overnight.

Table. Material and dilution ratio of WB

| number | Primary antibody | Dilution ratio | brand | Article number | secondary antibody |
| --- | --- | --- | --- | --- | --- |
| 1 | HSP70 | 1:1000 | abcam | ab181606 | Goat anti rabbit IgG(H&L) |
| 2 | Tsg101 | 1:1000 | abcam | ab125011 | Goat anti rabbit IgG(H&L) |
| 3 | CD63 | 1:200 | Santa | sc-5275 | Goat anti mouse IgG(H&L) |
| 4 | Calnexin | 1:500 | Proteintech | 10427-2 | Goat anti rabbit IgG(H&L) |

7) The next day, the membrane was taken out from 4 °C and incubated at room temperature for 30 minutes. Film washing: TBST film washing for 5 times, 3 minutes each time.

8) Secondary antibody incubation: the secondary antibody was diluted with 5% skim milk powder TBST, Goat anti rabbit IgG (H & L) HRP, 1:10000; Goat anti mouse IgG (H & L) HRP, 1:10000, gently shake at room temperature for 40 minutes. Film washing: TBST film washing for 6 times, 3 minutes each time.

9) After the ECL is added to the film, the reaction time is 3- 5 minutes, and the film is medium pressure in the dark room. The film is exposed for 10 seconds- 5 minutes (the exposure time is adjusted with different light intensities), developed for 2 minutes, and fixed.

10) Film scanning.
